# Efficacy and Molecular Mechanism of Quercetin on Constipation Induced by Berberine via Regulating Gut Microbiota

**DOI:** 10.3390/ijms25116228

**Published:** 2024-06-05

**Authors:** Mengyao Cui, Ying Li, Tingting Zheng, Huan Chen, Jinrui Wang, Yifan Feng, Hanyi Ye, Zhengqi Dong, Geng Li

**Affiliations:** 1 State Key Laboratory for Quality Ensurance and Sustainable Use of Dao-di Herbs, Institute of Medicinal Plant Development, Chinese Academy of Medical Sciences, Peking Union Medical College, Beijing 100193, Chinayli@implad.ac.cn (Y.L.);; 2 School of Pharmacy, Jiangxi University of Chinese Medicine, Nanchang 330004, China; 3 Key Laboratory of Bioactive Substances and Resources Utilization of Chinese Herbal Medicine, Ministry of Education, Chinese Academy of Medical Sciences, Peking Union Medical College, Beijing 100094, China; 4 Key Laboratory of New Drug Discovery Based on Classic Chinese Medicine Prescription, Beijing 100700, China; 5 Beijing Key Laboratory of Innovative Drug Discovery of Traditional Chinese Medicine (Natural Medicine) and Translational Medicine, Beijing 100700, China; 6 Institute of Traditional Chinese Medicine, Chinese Academy of Traditional Chinese Medicine, Beijing 100070, China

**Keywords:** *Amomum villosum* Lour. (AVL), berberine (BBR), quercetin (QR), compatibility of traditional Chinese medicine, constipation

## Abstract

Berberine (BBR) is used to treat cancer, inflammatory conditions, and so on. But the side effects of BBR causing constipation should not be ignored. In clinical application, the combination of *Amomum villosum* Lour. (AVL) and BBR can relieve it. However, the effective ingredients and molecular mechanism of AVL in relieving constipation are not clear. A small intestine propulsion experiment was conducted in constipated mice to screen active ingredients of AVL. We further confirmed the molecular mechanism of action of the active ingredient on BBR-induced constipation. Quercetin (QR) was found to be the effective ingredient of AVL in terms of relieving constipation. QR can efficiently regulate the microbiota in mice suffering from constipation. Moreover, QR significantly raised the levels of substance P and motilin while lowering those of 5-hydroxytryptamine and vasoactive intestinal peptide; furthermore, it also increased the protein expression levels of calmodulin, myosin light-chain kinase, and myosin light chain. The use of QR in combination with BBR has an adverse effect-reducing efficacy. The study provides new ideas and possibilities for the treatment of constipation induced by BBR.

## 1. Introduction

Constipation is a prevalent ailment that is typified by infrequent or challenging bowel motions. It is broadly categorized into functional constipation, organic constipation, and drug-induced constipation (DIC). The prevalence of DIC is high, and, according to surveys, about 10% to 15% of people will experience DIC, which is especially common in patients with chronic illnesses who have been taking medications for a long time. DIC will not only affect the life quality of the patient [1], but also influence how well the patient absorbs medication, which influences how effective the medication is [2], and even further leads to anal fissure, hemorrhoids, intestinal obstruction, and other serious complications [3]. Early intervention for DIC is essential. It is worth exploring effective treatment methods for DIC.

BBR is an effective monomer component of Chinese medicine in *Coptis chinensis* Franch [4], which is mainly extracted from the rhizome of *Coptis chinensis* Franch. As a traditional Chinese medicine ingredient, BBR has anti-inflammatory, antibacterial, and antiviral pharmacological activities, and is mainly used for the treatment of colds, enteritis, hepatitis, and other diseases. Although BBR has many medicinal effects, there are some possible side effects, among which digestive side effects are the most common, which may cause nausea, vomiting, stomach upset and other digestive reactions. Clinical studies have shown that long-term application of BBR will bring side effects of constipation [5,6]. It is also commonly used in animal experiments as a modeling drug for constipation models. Some studies have shown the possible mechanism of constipation is to inhibit the contraction of gastrointestinal smooth muscle by inhibiting the function of smooth muscle myosin [7], which in turn prolongs the retention time of intestinal contents and reduces the frequency of intestinal peristalsis [8]. In addition, BBR can inhibit gastric acid secretion, affecting the digestion of food and increasing the risk of constipation [9]. Therefore, the problem of constipation caused by BBR is gradually being emphasized and urgently needs to be solved.

*Amomum villosum* Lour. (AVL) is acrid and warm [7] and belongs to the ginger family. The main place of origin is the southern region of China [10]. Its fruit is widely used to treat digestive disorders. AVL can lighten the spleen and gastric symptoms, eliminate distension, and strengthen gastric activity [11]. The medicinal active ingredients of AVL are composed of volatile and nonvolatile ingredients. Common volatile ingredients include borneol (Bor), camphor (CAMP), bornyl acetate (BA), etc. Common nonvolatile ingredients include catechins (Cat), QR, etc. Research has proved that AVL can increase the peristalsis of the small intestine, which is beneficial to improve gastrointestinal function [12]. It is an effective herbal medicine to relieve constipation.

AVL and BBR are often used in combination for the treatment of gastrointestinal diseases, diabetes, and cardiovascular diseases. BBR has the effect of clearing heat, is an anti-inflammatory [13], and acts in suppressing pain [14], and AVL has the effect of promoting gastric peristalsis and gastric emptying [15]. BBR lowers blood sugar [16], and AVL promotes insulin secretion, effectively controlling blood sugar [17]. BBR lowers blood lipids [18], and AVL improves the function of the heart [19]. The use of them in combination can not only synergize the effect, but also effectively alleviate the constipation side effect caused by BBR, which has the effect of increasing the efficacy and reducing the adverse effects. However, the effective constipation-relieving components of AVL have not yet been clearly defined, and the molecular mechanism of relieving constipation induced by BBR has to be explored. BBR alters the composition of the gut microbiota [20]. Not all effects of BBR on the gut microbiota during treatment are beneficial. This includes a decrease in some probiotic bacteria such as the butyric acid-producing bacterium *Roseburia* and an increase in some conditionally pathogenic bacteria such as *Aspergillus* and *Streptococcus* [21]. Constipation is often a manifestation of gut microbiota imbalance. Some studies have shown that gut microbiota can regulate intestinal cell maturation and function [22]. The gastrointestinal metabolites short-chain fatty acids (SCFAs) are able to stimulate an increase in calcium ions in colonic L-cells via G-protein-coupled receptors, such as FFAR2 and FFAR3, which in turn promotes the secretion of the gastrointestinal hormone [23]. Gut microbiota also influences water and electrolyte balance, and studies have shown that *Lactobacillus* can influence the opening and closing of potassium and magnesium channels in the gut, thereby regulating electrolyte balance [24]. Gut microbiota also influences gastrointestinal motility function. It has been found that alterations in gut microbiota can regulate the intestinal barrier function by affecting the activity of the CAM-MLCK pathway [25]. Not only that, but the gut microbiota plays a crucial role in preserving the homeostasis of the enteric nervous system, and microbiota dysbiosis can reduce neuronal densities, change neuronal subtypes, and alter electrophysiological function. Both the volatile and nonvolatile ingredients of AVL can regulate gut microbiota and restore gastrointestinal health. In addition, research indicates that QR, a non-volatile component of AVL, has a significant impact on the diversity and composition of the gut microbiota. A study on antibiotic-treated mice showed that QR supplementation notably improved the diversity of the gut bacterial community. It also helped recover the intestinal barrier function, as evidenced by a decrease in serum d-lactic acid and serum diamine oxidase activity. Additionally, QR treatment led to an increase in the length of intestinal villi and mucosal thickness, along with enhanced butyrate production in feces [20].

**Scheme 1:**
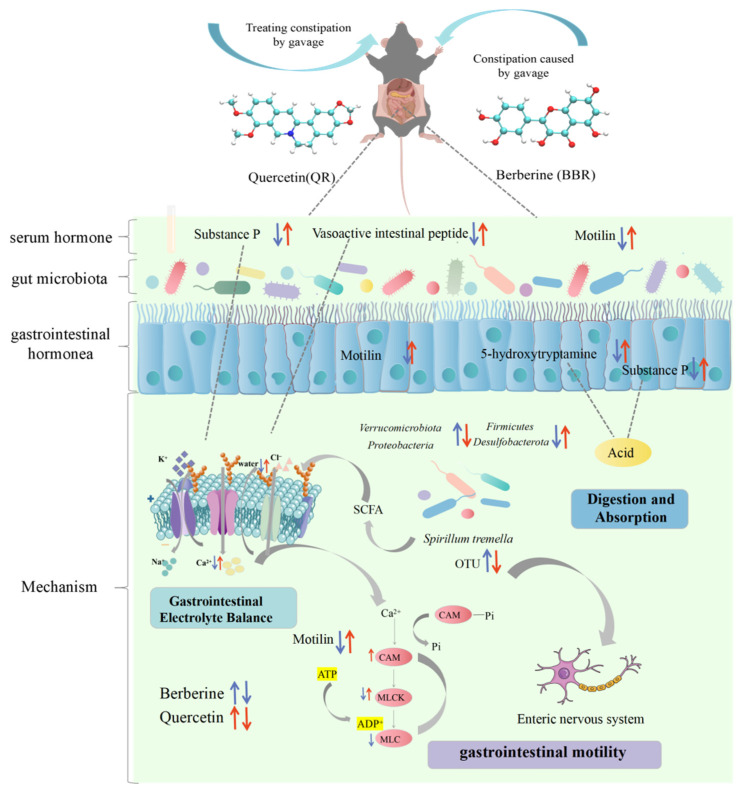
Mechanism of action of quercetin, an active ingredient of *Amomum villosum* Lour., on berberine-induced constipation. Note: Blue arrows indicate the regulatory trend of berberine and red arrows indicate the regulatory trend of quercetin. The mechanism of efficacy was assessed in terms of indicators related to gastrointestinal hormones, the gut microenvironment, and gastrointestinal smooth muscle peristaltic signaling pathways. Quercetin can relieve constipation by promoting digestion and absorption of gastrointestinal tract contents, promoting gastrointestinal tract water and electrolyte balance, and promoting gastrointestinal tract peristalsis.

This study aims to screen out the small molecules of herbs in constipation alleviation, and explore the efficacy and molecular mechanism of constipation alleviation by regulating gut microbiota, which provides new ideas and a basis for reducing the toxic and side effects of BBR and expanding its clinical application in combination. The mechanism of action of quercetin on berberine-induced constipation is demonstrated in Scheme 1.

## 2. Results

### 2.1. Screening of Active Ingredients in AVL for Constipation Relief

**Figure 1:**
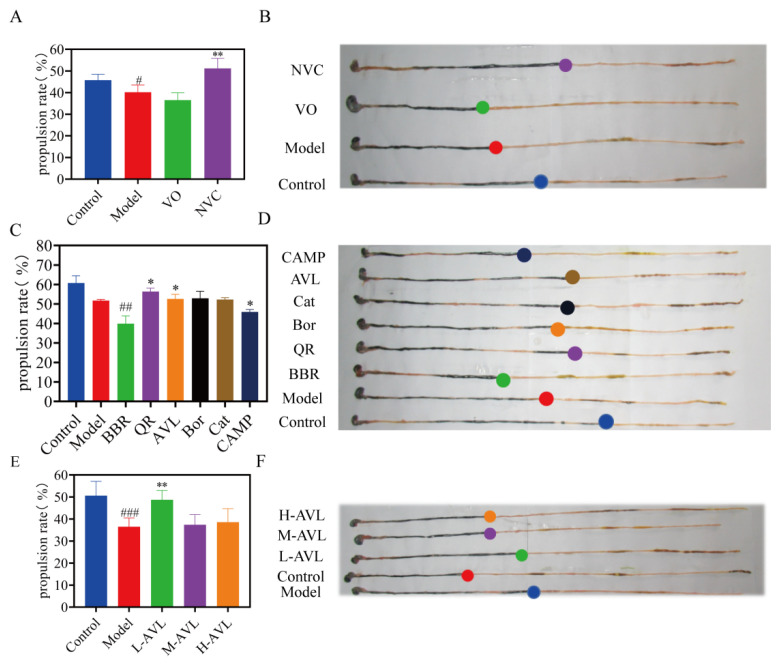
Carbon powder propulsion diagram. (**A**) Carbon powder propulsion diagram of volatile and nonvolatile ingredients of AVL. (**B**) Histogram of carbon powder propulsive rate of volatile and nonvolatile ingredients of AVL (mean ± SD, *n* = 6). (**C**) Carbon powder propulsion diagram of each component of AVL. (**D**) Histogram of carbon powder propulsion rate of each component of AVL (mean ± SD, *n* = 6). (**E**) Carbon powder propulsive diagram of AVL with different doses. (**F**) Histogram of carbon powder propulsion rate of different doses of AVL (mean ± SD, *n* = 6). Significance was assessed by using *t*-test vs. Control group, ^#^ *p* < 0.05, ^##^ *p* < 0.01, ^###^ *p* < 0.001 vs. Model, * *p* < 0.05, ** *p* < 0.01.

The variations in the intestinal propulsion rates are illustrated in Figure 1. Mice in the Control group had a considerably higher intestinal propulsion rate than those in the Model group (*p* < 0.05). It proves that the mouse constipation model was built effectively. Following the intervention, there was a statistically significant (*p* < 0.05) increase in the nonvolatile group’s intestinal propulsion rate. The nonvolatile group’s intestinal propulsion rate was 51.17 ± 4.64%, greater by 10.97 ± 7.97% than that of the Model group (Figure 1A,B). These findings suggested that nonvolatile components in AVL might relieve constipation by promoting peristalsis and accelerating the transit of activated carbon through the small intestine. In the meantime, the outcomes of studies on small intestine propulsion not only suggested that QR could relieve constipation by promoting peristalsis and accelerating the passage of activated charcoal through the small intestine, but also indicated that BBR and CAMP increased constipation in mice (Figure 1C,D). Finally, the results also showed that an AVL dose of 70 mg·kg^−1^ can effectively relieve constipation caused by BBR (Figure 1E,F).

### 2.2. Efficacy of Different Doses of QR in Relieving Constipation

#### 2.2.1. Effects of Different Doses of QR on the Body Weights of Mice

**Figure 2:**
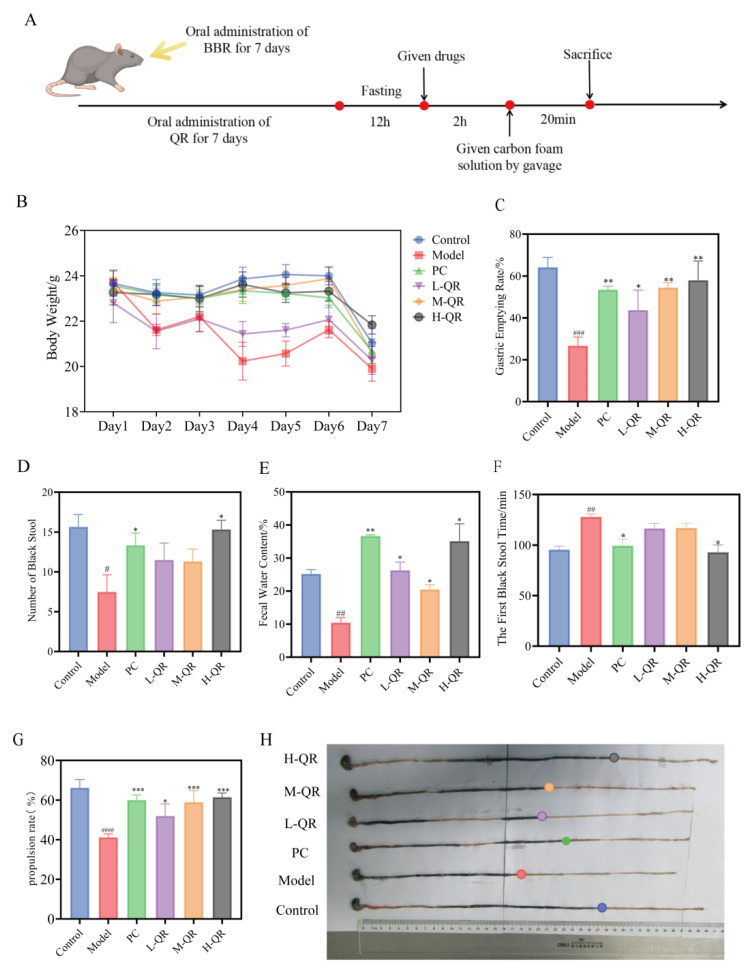
(**A**) Flow charts of different QR doses by gastric perfusion. (**B**) Effects of different doses of QR on body weights of mice for 7 d (mean ± SD, *n* = 6). (**C**) Histogram of mice gastric emptying rate in different QR dose groups (mean ± SD, *n* = 6). (**D**) Histogram of the number of black stool occurrences in different QR dose groups (mean ± SD, *n* = 6). (**E**) Histogram of mice fecal water contents in different QR dose groups (mean ± SD, *n* = 6). (**F**) Histogram of the first black stool time in different QR dose groups (mean ± SD, *n* = 6). (**G**) Histogram of mice intestinal propulsive rate in different QR dose groups (mean ± SD, *n* = 6). (**H**) Intestinal propulsion of carbon powder in different QR dose groups. Significance was assessed by using *t*-test vs. Control group, ^#^ *p* < 0.05, ^##^ *p* < 0.01, ^###^ *p* < 0.001, ^####^ *p* < 0.0001 vs. Model, * *p* < 0.05, ** *p* < 0.01, *** *p* < 0.001.

Throughout the course of the administration period, the body weights of the constipated mice were measured every day in order to assess the impact of QR on changes in body weight. Due to the pharmacological effect of BBR [26], the bodies of the mice in the Control group had reduced fat *(p* < 0.05). After the treatment intervention, the body weights in the Positive Control, Middle-QR, and High-QR groups were not significantly different from those in the Control group. It can be seen that Middle-QR and High-QR doses can effectively alleviate the BBR-induced abnormal weight loss in mice and restore the body weight of mice, as weights in the Model group decreased significantly compared to those in the normal levels (Figure 2B).

#### 2.2.2. Fecal Water Content of Mice

Fecal water content is an important indicator of the success of constipation model construction. Dry feces will increase the burden of defecation. The increase in water content indicates relief of constipation. In addition, the shorter the time it takes for the first black stool to be discharged, the faster the peristalsis throughout the intestines and the greater the intestinal transportation capacity. The number of black stools in a certain period of time can also determine the degree of constipation.

As shown in Figure 2, mice with constipation showed constipation symptoms such as reduced fecal water content, prolonged appearance of black stools, and reduced black-stool elimination (*p* < 0.05). After treatment, the mice showed varying degrees of constipation relief (Figure 2D–F).

The results demonstrated that QR was effective in increasing fecal water content and promoting fecal elimination. The time to expel black stools was fastest in the High-QR group and was closer to the Control group than to the Positive Control, Low-QR, and Middle-QR groups (*p* < 0.05). The results of the number of black stools expelled in six hours, as shown, suggested that the High-QR dose had a better effect on relieving constipation, and its efficacy was closer to that of the Positive Control group.

#### 2.2.3. Effects of Different QR Doses on Gastric Emptying Rate and Intestinal Propulsive Rate

Mice exposed to QR demonstrated a remarkable gastric-emptying rate increase compared with mice exposed to BBR (*p* < 0.001), which was suggestive of a consolidated potency of QR in promoting gastric motility (Figure 2C). The small intestinal propulsion rates of mice in the Control group was 66.20 ± 4.17%, while that of the Model group was 41.23 ± 1.70% (Figure 2G,H), which was significantly reduced (*p* < 0.001). The different QR doses significantly increased the small intestinal propulsion rate in mice. As compared to the Low-dose QR group, the Middle- and High-dose QR groups could better relieve constipation, showing a dose-dependent efficacy.

### 2.3. Mechanism of QR in Alleviating Constipation

#### 2.3.1. Effect of QR on the Gut Microbial Diversity

**Figure 3:**
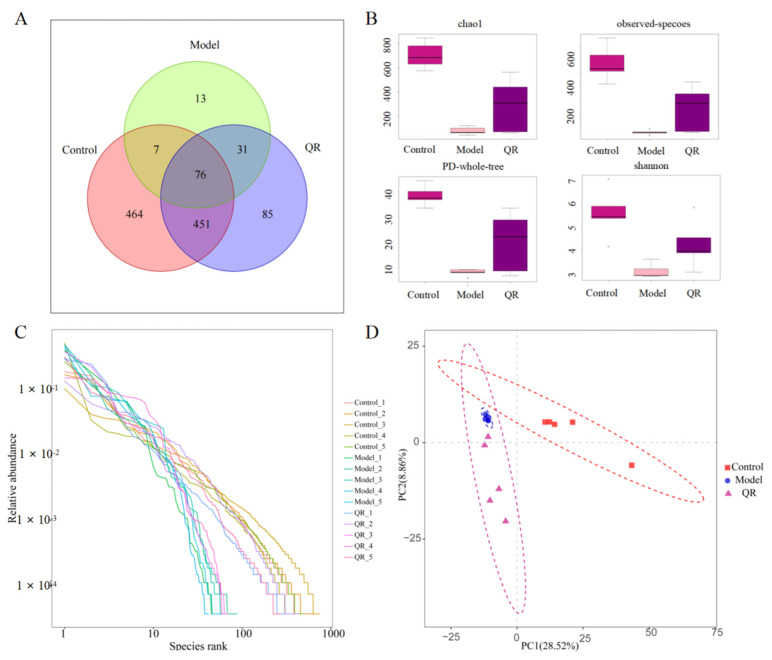
Effects of QR on the diversity of gut microbiota. (**A**) Venn diagram of OTU quantity. (**B**) Alpha diversity index box plots (*n* = 5). (**C**) Rank abundance diagram. (**D**) OTU-based Partial Least Squares Discriminant Analysis. The dose of the QR group was 280 mg·kg^−1^. Significance was assessed by using *t*-test.

The Venn diagram presents a breakdown of the common and unique operational taxonomic unit (OTU) numbers among each group (Figure 3A). BBR significantly reduced the number of OTUs in mice gut microbiota, which were effectively restored by QR. This indicated that BBR reduced the diversity of gut microbiota and caused constipation, while QR relieved constipation by increasing the diversity of gut microbiota in mice.

One significant metric that represents the quantity, uniformity, and variety of gut microbiota is alpha (α) diversity. The findings presented (Figure 3B) indicate that feeding mice with BBR significantly reduced the abundance and diversity of gut microbiota in the mice. Specifically, the Observed-species, Chaol, PD-whole-tree, and Shannon indices of the Model group were significantly lower (*p* < 0.05) compared with the Control group. Conversely, the QR groups exhibited a statistically significant difference from the Model group (*p* < 0.05), indicating that the application of QR improved and restored the diversity and richness of the gut microbiota that had been compromised by the mice’s BBR diet.

The examination of beta diversity revealed that the Model group’s gut microbiota had more homogeneity and aggregation than the Control group’s (Figure 3C,D), clearly demonstrating the impact of BBR on the gut microbiota composition of mice. Given the wide gap between the QR group and the Model group, it was possible that QR intervention could partially restore the gut microbiota’s structural diversity and balance.

#### 2.3.2. Effects of QR on the Composition of Mice Gut Microbiota

**Figure 4:**
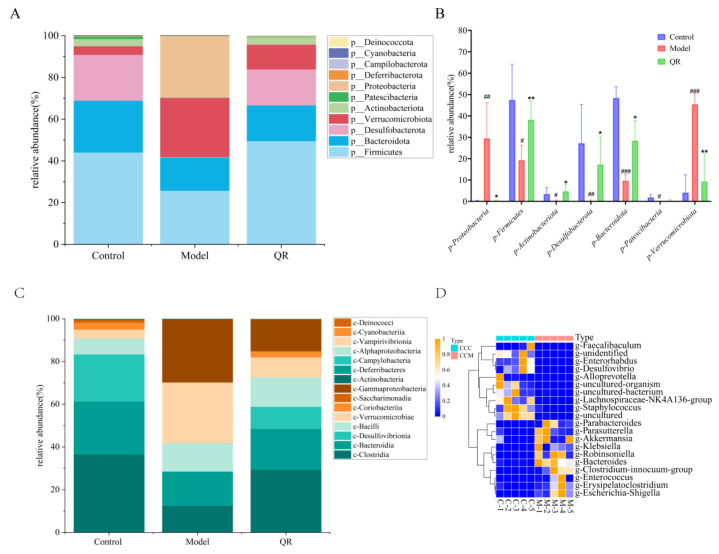
Effects of QR on species composition of microbiota. (**A**) Composition of gut microbiota at the phylum level in different mice groups. (**B**) Histogram of relative abundance of gut microbiota at the phylum level (*n* = 5). (**C**) Composition of gut microbiota at the class level in different mice groups. (**D**) Heatmap analysis. The dose of the QR group was 280 mg·kg^−1^. Significance was assessed by using *t*-test. Control group, ^#^ *p* < 0.05, ^##^ *p* < 0.01, ^###^ *p* < 0.001. vs. Model, * *p* < 0.05, ** *p* < 0.01.

At the phylum level, the relative abundances of gut microbiota in each group were analyzed. *Firmicutes* and *Bacteroidota* were the dominant and main phyla of the mice gut microbiota in each group [27], followed by *Desulfobacteria*, *Proteobacteria*, *Verrucomicroblota*, etc. (Figure 4A). As compared to the Control group, BBR decreased the relative abundance of *Firmicutes* and increased that of *Proteobacteria*, which were significantly restored by QR (Figure 4B). *Firmicutes* relieve symptoms of low gastrointestinal capacity such as constipation, gastrointestinal ulcers, and the postoperative period [28]. *Proteobacteria* are pathogenic Gram-negative bacteria that cause inflammatory diseases [29]. *Verrucomicrobia* can cause metabolic inflammation. Both are pathogenic and affect normal gastrointestinal physiological functions [30]. The composition of mice gut microbiota in each group at the genus level was analyzed based on the composition information of the annotated species table, and the distribution of gut microbiota was drawn using the R package. As compared to the Control group, the relative abundances of beneficial bacterial genera *Lachnospiraceae_NK4A136_group*, *Dubosiella,* and *Lachnoclostridium* in the Model group decreased, while those of pathogenic bacterial genera *Parabacteroides* and *Escherichia-Shigella* increased. The abundance of bacteria in the QR group was closer to that in the Control group (Figure 4C).

**Figure 5:**
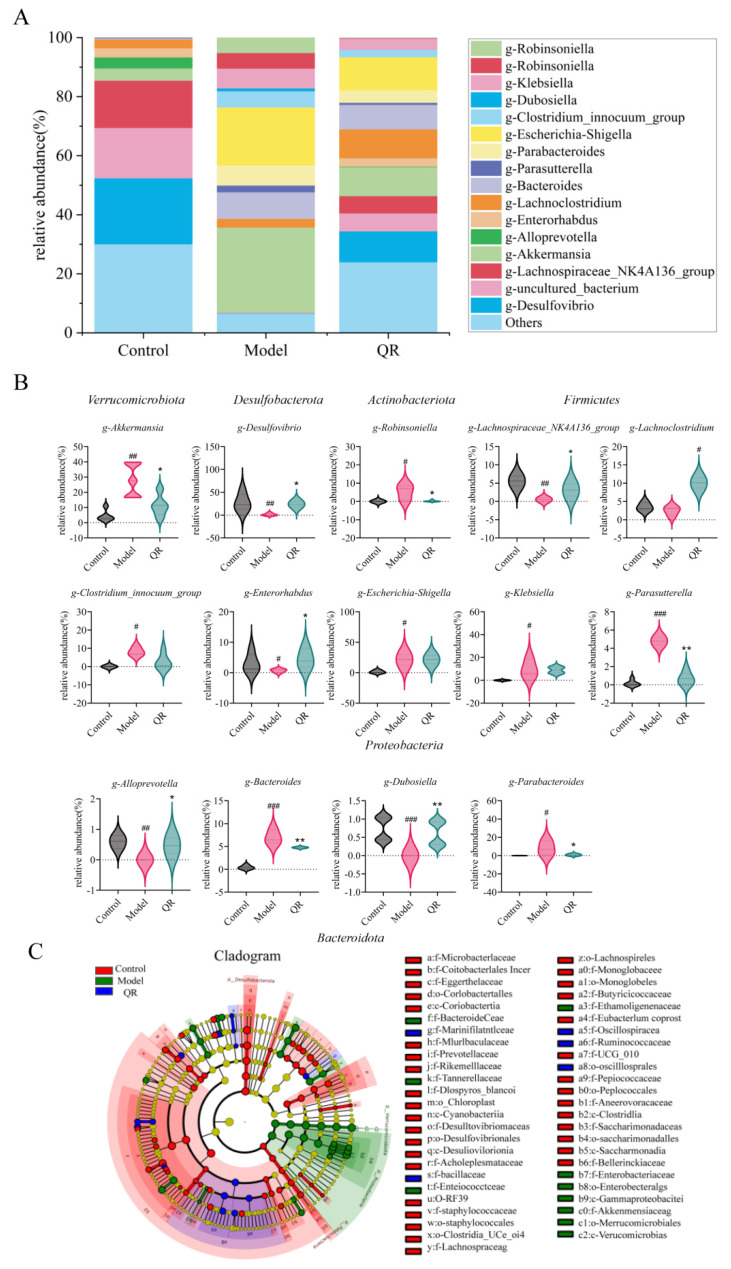
Effects of QR on species composition of microbiota. (**A**) Composition of gut microbiota at the genus level in different mice groups. (**B**) Statistical chart of violin type of gut microbiota at the genus level in different mice groups. (**C**) Linear discriminant analysis effect size. The dose of the QR group was 280 mg·kg^−1^. Significance was assessed by using *t*-test. Control group, ^#^ *p* < 0.05, ^##^ *p* < 0.01, ^###^ *p* < 0.001. vs. Model, * *p* < 0.05, ** p < 0.01.

LEfSe analysis showed that there were 27 species from phylum to genus showing significant differences (Figure 5C). QR promoted the enrichment of *Oscillospirales*. At the family level, the dominant bacteria in the Control, Model, and QR groups were *Desulfovibrionaceae* and *Microbacteriaceae*, *Enterobacteriaceae* and *Akkermansiaceae*, and *Bacillaceae* and *Oscillospiraceae*, respectively. *Oscillospirales* is the dominant bacterial group in the QR group, which is a probiotic that promotes the digestion and absorption of nutrients, promotes growth, and strengthens the immune system [31].

BBR is bitter in flavor and cold in nature. The abnormal elevation of the abundance of cold-related key flora such as *Proteobacteria*, *Escherichia-Shigella*, and *Verrucomicroblota* [32] after the administration of BBR proved that BBR led to the development of cold-type constipation in normal mice. QR, which is warm in nature, can effectively neutralize the cold nature of BBR. It not only reversed the abnormal elevation of relative abundance of cold-related flora, but also significantly elevated the relative abundance of heat-related key flora such as *Bacteroidota* and *Lachnoclostridium* [33]. The results demonstrated that QR can be used to improve stools and defecation caused by spleen and stomach weakness (Figure 4B,D).

#### 2.3.3. Effects of QR on Gastrointestinal Hormones in Mice

**Figure 6:**
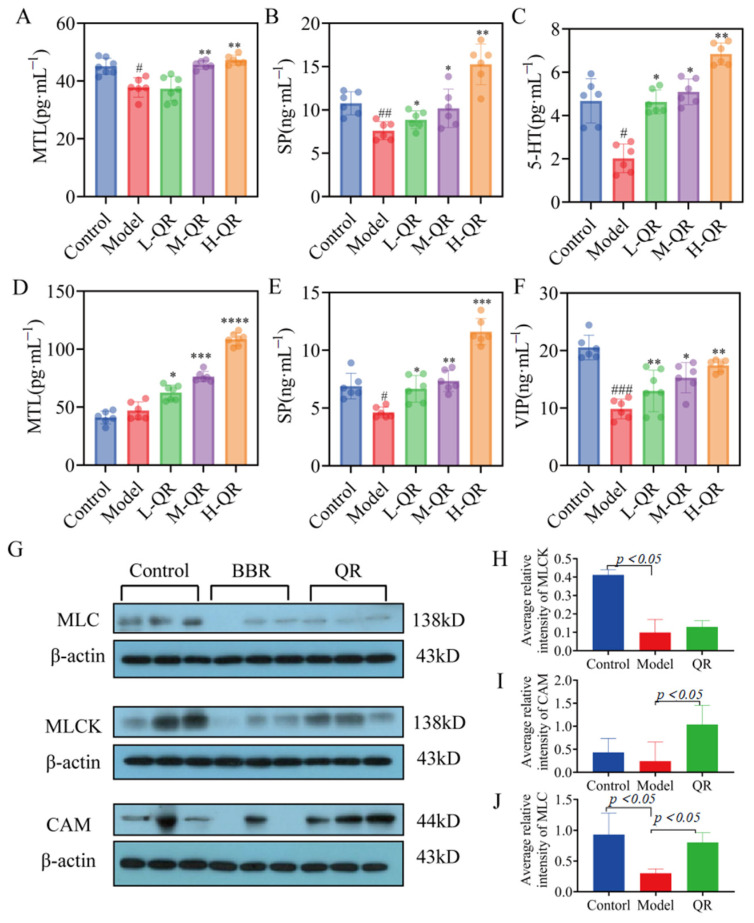
(**A**) Histogram of 5-HT level in mouse stomachs (mean ± SD, *n* = 6). (**B**) Histogram of MTL level in mouse stomachs (mean ± SD, *n* = 6). (**C**) Histogram of SP level in mouse stomachs (mean ± SD, *n* = 6). (**D**) Histogram of MTL level in serum of mice (mean ± SD, *n* = 6). (**E**) Histogram of serum SP level in mice (mean ± SD, *n* = 6). (**F**) Histogram of VIP level in mice (mean ± SD, *n* = 6). (**G**) Western blot analysis of the expression levels of CaM-MLCK pathway-related proteins in different mice groups. (**H**) Expression level of MLCK in the small intestine of mice. (**I**) CAM expression level in the small intestine of mice. (**J**) MLC expression level in the small intestine of mice. Significance was assessed by using *t*-test. Control group, ^#^ *p* < 0.05, ^##^ *p* < 0.01, ^###^ *p* < 0.001. vs. Model, * *p* < 0.05, ** *p* < 0.01, *** *p* < 0.001,**** *p* < 0.0001.

Gastrointestinal hormones are important substances that regulate intestinal motility, the secretion of digestive juices, and absorption of nutrients, etc. They not only regulate the level of peristalsis in the gastrointestinal tract, but also regulate gastrointestinal water and electrolyte balance. For example, MTL can improve gastrointestinal water and electrolyte transportation [34], and patients with long-term constipation have significantly reduced levels of MTL [35,36]. VIP can regulate the intestinal metabolism of water and electrolytes and increase the release of Cl^−^, HCO^3−^, and water [33]. SP is an excitatory neurotransmitter [34], which can contract the smooth gastrointestinal muscles and promote intestinal peristalsis and gastric discharge [37,38]. Studies have shown that the release of SP from nerve endings can activate platelets to release 5-HT [28], which is a key neurotransmitter in the brain–gut axis [39]. About 90% of 5-HT in the human body is synthesized by the intestine and distributed in the intestinal chromaffin cells, which are involved in regulating colon secretion, intestinal movement, and the gastrointestinal sensory function. As shown in Figure 6, the levels of excitability neurotransmitters (MTL, VIP, SP, and 5-HT) of the Model group were significantly decreased compared to the Control group (*p* < 0.05), which was reversed by QR treatment (*p* < 0.005). The results showed that QR, especially at high doses, enhanced serum and gastric excitatory factors and reversed the inhibitory effect of BBR on gastrointestinal motility (Figure 6A–E).

#### 2.3.4. Effects of QR on CAM-MLCK Signaling Pathway in the Small Intestine of Mice

The CAM-MLC signaling pathway regulates smooth muscle contraction and plays a key role in dynamic signaling in gastrointestinal smooth muscle cells. Western blot analysis was used to determine the expression of CAM in the small intestine smooth muscle, and the results showed that the expression of this protein in constipated mice increased with stimulation with QR (Figure 6G). In addition, QR significantly reversed the decreased expression of MLC protein in the small intestine of constipated mice (Figure 6H–J).

#### 2.3.5. Biological Safety of QR of Different Doses

**Figure 7:**
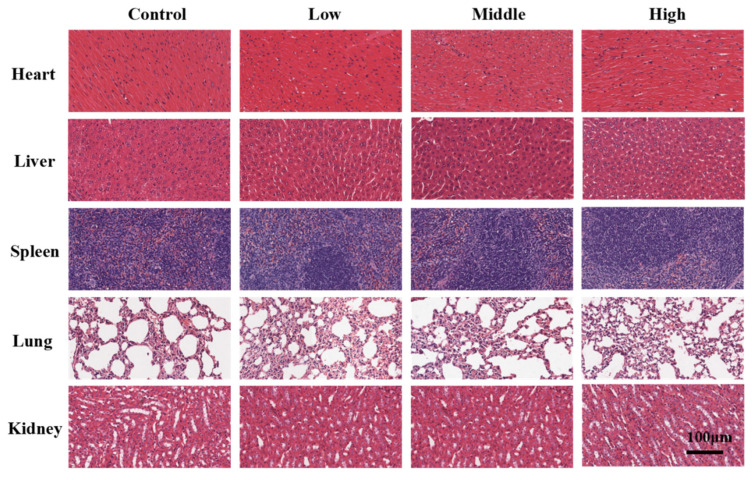
Histological sections of the organs of different QR dose groups.

**Figure 8:**
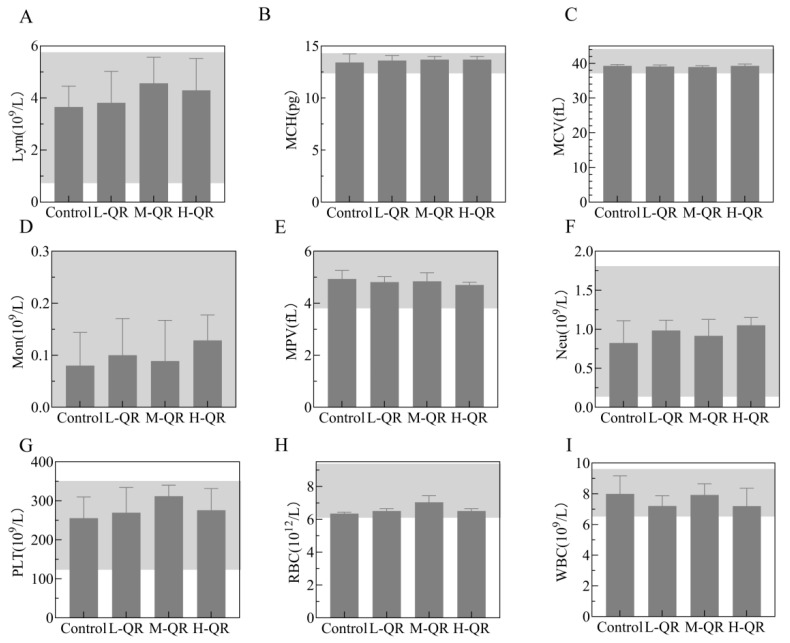
The effects of different QR doses on blood routine indices in mice (mean ± SD, *n* = 6). (**A**) WBC. (**B**) RBC. (**C**) PLT. (**D**) MCV. (**E**) MCH. (**F**) MPV. (**G**) Lym. (**H**) Neu. (**I**) Mon. Significance was assessed by using *t*-test.

The immunohistochemistry of tissue sections showed that, as compared to the Control group, there was no abnormal injury and inflammation in the mice exposed to QR with different doses (Figure 7 and Figure 8). Histopathological abnormalities were also not observed in organs. Routine blood analysis was performed to further quantify the toxicology of QR. The routine blood indices, including WBC, MVC, RBC, MPV, MCH, PLT, Lym, Neu, and Mon, were measured. The continuous intragastric administration of different QR doses for 7 days resulted in slight fluctuations in all the analytical test values as compared to those of the Control group; however, all values were within the normal range, showing no abnormalities.

## 3. Discussion

BBR is frequently used to treat various diseases such as intestinal infections, high cholesterol, and diabetes. However, its application is hampered by side effects, particularly constipation. This directly impacts patient adherence to the medication, thereby affecting its therapeutic effectiveness. In our study, the mouse constipation model was established by the intragastric administration of BBR (130 mg·kg^−1^). The mice showed clinical symptoms of constipation, such as poor mental state, reduced food intake, weight loss, small stool size, and dryness [40]; however, they did not die [41]. We discovered that BBR slows food digestion and results in gastrointestinal dysfunction by inhibiting the release of pro-digestive hormones. While BBR has commendable antibacterial properties, it also suppresses the normal gut flora, thereby affecting intestinal peristalsis and causing stool retention. Furthermore, BBR destabilizes the normal levels of hormones both in serum and the stomach, leading to disruptions in the gastrointestinal tract’s electrolyte balance. This influences stool composition and motor nerve conduction, and exacerbates difficulties in bowel movements.

QR, the small molecules of herbs, for relieving constipation and could effectively ease DIC induced by BBR. QR can effectively promote the digestion and absorption of gastrointestinal contents. Gastrointestinal hormones such as SP and 5-HT stimulate gastric acid secretion. Not only does gastric acid activate digestive enzymes for further food digestion, but it also stimulates the stomach muscles to contract and induce peristalsis. The normal gut microbiota helps the digestion and absorption of food in the gut. The abundance of *Firmicutes* is closely related to gastrointestinal peristalsis [42], especially *Lactobacillus*, *Ruminococcus*, and *faecium* species, which can significantly improve the imbalance of gastrointestinal motility caused by constipation, diarrhea infection, and surgery [43]. Most of the species in the phylum *Proteobacteria* are Gram-negative pathogenic bacteria. An increase in their abundance is associated with inflammatory diseases [29]. In general, *Proteobacteria* are used as criteria to identify disorders and potential diseases related to gut microbiota. Significant changes in the abundance of *Verrucomicrobia* species are associated with diseases, such as nonalcoholic fatty liver disease and metabolic inflammation [44]. In this study, BBR decreased the relative abundance of *Firmicutes* and increased that of Proteus and *Verrucomicrobia*, which led to constipation. QR effectively manages the balance of beneficial and harmful microbiota in the gut to aid in food digestion and absorption.

QR can effectively balance the water and electrolyte balance, increase the fecal water content, and relieve constipation. VIP can regulate the intestinal metabolism of water and electrolytes and increase the release of Cl^−^, HCO^3−^, and water [33]. Some research suggests that SP primarily regulates intestinal secretion in the gastrointestinal tract. It promotes the release of Cl^−^, K^+^, and water from intestinal epithelial cells by stimulating the Neurokinin 1 (NK1) receptor, which subsequently alters the gut’s electrolyte balance [45]. Moreover, SP can influence smooth muscle cells through Neurokinin 2 (NK2) receptors, reducing Na^+^ uptake and thus modifying electrolyte levels [46]. As the dominant bacterial species in the mice gut microbiota of the QR group, Spirillum tremella can ferment sugar and starch into short-chain fatty acids [30]. Short-chain fatty acids such as butyric, propionic, and acetic acids can release Cl^−^ and water by stimulating intestinal cells. Together, QR increased the fecal water content by significantly increasing some gastrointestinal hormone content in mice with constipation and regulating intestinal flora and its metabolic activities, repairing BBR-induced gastrointestinal water and electrolyte disturbances.

Promoting gastrointestinal motility is an important way for QR to relieve BBR-induced constipation. Ca^2+^ is an important electrolyte for promoting gastrointestinal motility. Both gastrointestinal hormones 5-HT and VIP increase intracellular Ca^2+^ concentrations. In order to explore the mechanism of action, the classical signaling pathway which regulates gastrointestinal smooth muscle, CAM-MLCK, was selected as the breakthrough point [47,48]. Western blot analysis was used to detect protein expression levels of MLCK, MLC, and MLCK signaling factors in the mice’s gastrointestinal tract. The CAM-MLC signaling pathway regulates the contraction of smooth muscles and plays a key role in the dynamic signal transduction of smooth muscle cells in the gastrointestinal tract. The complex formed by CAM and Ca^2+^ can further activate MLCK, which phosphorylates MLC to generate ATP, thereby achieving smooth muscle contraction. BBR inhibited smooth muscle contraction by inhibiting the expression of signaling proteins. QR significantly promoted the expression of the CAM-MLC signaling pathway and alleviated constipation. In addition to the CAM-MLCK pathway, QR has the potential to promote gastrointestinal neurotransmission by restoring gut microbiota abundance. The gut microbiota plays a crucial role in preserving the homeostasis of the enteric nervous system, and microbiota dysbiosis can reduce neuronal densities, change neuronal subtypes, and alter electrophysiological function. It has been shown that the microbiota in the mouse intestine can influence intestinal motility, and that impaired function of the enteric nervous system and disturbances in intestinal physiology are present in germ-free mice [49]. However, recolonizing for germ-free mice can return the gut physiology and enteric nervous system to normal levels [50]. This shows the extent to which the microbiota and its abundance affect the enteric nervous system. The analysis of gut microbiota in the BBR group showed a significant decrease in their diversity. QR could alleviate constipation by increasing the diversity of gut microbiota in mice. QR administration improved the intestinal function by regulating the gut microbial microenvironment.

## 4. Materials and Methods

### 4.1. Materials

*Amomum villosum* Lour. (AVL) was acquired from Tongrentang Co., Ltd. (Lot No. 22012301, Beijing, China). Quercetin (QR) was acquired from Ruifen Biotechnology Co., Ltd. (Lot No. RFS-H00911809026, Chengdu, China). Borneol (Bor) was acquired from Delta biological technology Co., Ltd. (Lot No. RM02200906, Chengdu, China). Berberine (BBR) was acquired from Ronghe Pharmaceutical Technology Development Co., Ltd. (Lot No. 220326, Shanghai, China). Camphor (CAMP) was acquired from Delta biological technology Co., Ltd. (Lot No. RM02200905, Chengdu, China). Catechins (Cat) were acquired from Zelang Biotechnology Co., Ltd. (Lot No. GL20210325, Nanjing, China). Sodium carboxymethyl cellulose (CMC-NA) was acquired from Wanjia Shouhua Biotechnology Co., Ltd. (Beijing, China). Antibodies against CAM, MLCK and MLC were acquired from Tianzhengyuan Biotechnology Co., Ltd. (Wuhan, China). ELISA kits for 5-HT, VIP, SP, MTL were acquired from Jinenlai Biotechnology Co., Ltd. (Beijing, China).

### 4.2. Preparation of Drugs

The seeds of AVL were hulled and finely ground into a powder. We then boiled a specific measure of their powder in water and filtered it to acquire a clear liquid. The liquid underwent centrifugation, and the supernatant was subsequently collected for further utilization. The indigestible mixture was prepared, consisting of water, milk powder, sugar, and lard in equal proportions of 1 g each. The active ingredients were uniformly dispersed in water at designated concentrations, ensuring their thorough blending through the use of ultrasonication.

### 4.3. Animals and Treatment

Three hundred male C57BL/6J mice aged 6 weeks with body weight 18 g–20 g were provided by Beijing Huafukang Biotechnology Co., Ltd. (Beijing, China), and the animal experiment complied with the protocol of the Chinese Academy of Medical Sciences & Peking Union Medical College (No. SLXD-20220509013). The feeding temperature was 20 ± 2 °C, the humidity was 60 ± 5%, and the animals were allowed to drink freely under light and dark circulation for 12 h. Adaptive feeding for 3 days.

### 4.4. Effect of Volatile and Nonvolatile Ingredients of AVL on Constipation

Four different groups of mice were stochastically divided, including the Control group, Model group, Volatile group (Bor, CAMP and BA 40 mg·kg^−1^ each) [51], and Nonvolatile group (Cat and QR 60 mg·kg^−1^ each), each having ten mice. All groups, bar the Control group, were administered indigestible gavage mixture [52], twice daily, at a dosage of 10 μL·g^−1^ for three consecutive days. The mice in the drug treatment groups were administered their respective substances over the following four days.

One week later, the mice were fasted for 12 h. Post 30 min of drug administration, the mice were given a carbon powder suspension, dosed at 10 μL·g^−1^. Fifteen minutes thereafter, the mice were humanely euthanized, in line with ethical principles governing animal experimentation. The distance the carbon powder travelled in the small intestine was measured and recorded.

Carbon powder propulsion rate (%) = carbon powder propulsion length/total length of small intestine ×100% [53].

### 4.5. Effect of Different Components of AVL on Constipation

The mice were stochastically divided into eight groups, namely, the Control group, Model group, *Amomum villosum* Lour group (120 mg·kg^−1^), BBR group (140 mg·kg^−1^), Cat group (120 mg·kg^−1^), QR group (120 mg·kg^−1^), Bor group (120 mg·kg^−1^), and CAMP group (120 mg·kg^−1^), with 10 mice per group. The drug administration modeling process and the operation process for obtaining carbon powder propulsion rates were the same as those under Section 4.4.

### 4.6. Effect of Different Doses of AVL on Constipation

Five groups of mice were stochastically divided, namely, the Control group, Model group, Low-AVL group (120 mg·kg^−1^), Middle-AVL group (240 mg·kg^−1^) and High-AVL group (480 mg·kg^−1^) with 10 mice per group. Except the mice in the Control group, all mice were given BBR with a concentration of 130 mg·kg^−1^ for 6 days. The operation process for obtaining carbon powder propulsion rates was the same as those under Section 4.4. Different tissues were taken and preserved in 4% tissue fixative.

### 4.7. Effect of Different Doses of QR on Constipation

We randomly divided the mice into six groups, including the Control group, Model group, Positive Control group (bisacodyl, 100 mg·kg^−1^), Low-QR group (70 mg·kg^−1^), Middle-QR group (140 mg·kg^−1^), and High group (280 mg·kg^−1^) [54]. The drug administration modeling process and the operation process for obtaining carbon powder propulsion rates were the same as those under Section 4.4. The whole blood samples were taken to collect serum obtained by centrifugation. The fecal samples were obtained from the cecum to put into a freezing tube and stored at −80 °C [55]. The gastric and small intestinal tissues were taken and stored in a refrigerator at −80 °C. Different tissues were taken and preserved in 4% tissue fixative.

### 4.8. Determination of the Fecal Water Content

After administration, mice were kept separately in the feeding cage, underwent water fasting for 20 min, and were kept away from urine-soaked feces. Fresh fecal samples were collected in a centrifuge tube and weighed. The fecal samples were then kept in a constant-temperature air-drying oven to weigh. We calculated the fecal water content according to Equation.

Water content in feces (g) = (wet weight of feces − dry weight of feces)/wet weight of feces

### 4.9. Determination of the First Black Stool Time and the Number of Black Stool Occurrences in 6 h

After administration, the mice were gavage-administered carbon powder suspension, and the first black stool time and the number of black stool occurrences in 6 h were recorded from the time of carbon powder administration.

### 4.10. Determination of Gastric Emptying Rate and Intestinal Propulsive Rate

After 2 h of administration, the mice in each group were given the semi-solid paste with a dose of 15 mg·kg^−1^. Waiting for 20 min, the mice were sacrificed by cervical dislocation, followed by an opening of the abdomen and ligation of the gastric cardia and pylorus. The stomach was taken out, wiped with filter paper to dry it, and weighed to record its total weight. The stomach was then cut, and the contents were washed off. It was then dried with filter paper and weighed to record its net weight. Gastric emptying rate was recorded and calculated.

### 4.11. 16S rRNA Gene Sequencing and Analysis

Genomic DNA was extracted and its integrity was checked. The concentration and purity of genomic DNA were detected after extraction. The hypervariable variable regions of bacterial 16S rRNA gene were amplified with universal primers (338F 5′-ACTCCTACGGGAG-GCAGCAG-3′ and 806R 5′-GGACTACHVGGGTWTCTA-AT-3′) and sequenced using Illumina MiSeq platform. The raw sequence reads were quality filtered, followed by trimming, filtering, and removal of chimeras. Then, OTU clustering and annotation were performed. The OTU clusters were used for the alpha and beta diversity analyses, while the annotation results were used to obtain the classification information for each level. Furthermore, correlation analysis was performed to identify the differences in the composition and structure of gut microbiota among the groups.

### 4.12. ELISA

Using a homogenizer to crush and pulverize the stomach tissues, the supernatant was separated using centrifugation. ELISA kits (Wanjia Shouhua Biotechnology, Beijing, China) were used to measure the amounts of gastrointestinal hormones, including SP, 5-HT, MTL, and VIP, in the serum and stomach tissue of mice.

### 4.13. Western Blot

Protein expression levels in the gastrointestinal tract were analyzed by Western blotting. The tissues were homogenized using a tissue homogenizer (G100, Coyote, Beijing, China). The lysate was then centrifuged to obtain the supernatant. The protein concentration was adjusted using RIPA buffer. The same amount of sample protein was loaded onto gel and separated by electrophoresis. The proteins were then transferred to the membrane. The primary antibodies against CAM, MLCK, and MLC were diluted with 3% BSA-TBST and incubated, followed by washing with TBST. The secondary antibodies were diluted and incubated with the membrane. Enhanced chemiluminescence was added to the membrane. After exposure, the membrane was photographed directly. The integral optical density (IOD) value of the band was calculated.

### 4.14. H&E Analysis

The organs were immersed in the 4% formaldehyde fixative solution, paraffinized, and sectioned, followed by staining with hematoxylin and eosin. The mice tissue sections of each group were then observed and compared under a digital microscope (BX51, Olympus, Tokyo, Japan). The organs included liver, spleen, kidneys, heart, and lungs.

### 4.15. Whole Blood Analysis

The whole blood samples were taken and kept for 20 min at room temperature. The blood samples were shaken and mixed to avoid coagulation, hemolysis, and other conditions. Whole blood analysis was then performed using automatic blood cell analyzer (BC-5100, Mindray, Shenzhen, China), and the values were recorded.

### 4.16. Statistical Analysis

The data obtained from this experiment were summarized and classified, and the data were analyzed by *t*-test analysis of double tail distribution through statistical software SPSS 25.0. The results were expressed as Mean ± SD deviation. Significance was assessed by using *t*-test vs. Control group, ^#^ *p* < 0.05, ^##^ *p* < 0.01. vs. Model, * *p* < 0.05, ** *p* < 0.01.

## 5. Conclusions

In this study, the small molecules of AVL were investigated. The results showed that QR has the most prominent relieving effect on constipation. QR can relieve constipation by promoting digestion and absorption of gastrointestinal tract contents, promoting gastrointestinal tract water and electrolyte balance, and promoting gastrointestinal tract peristalsis. QR elevated gastrointestinal hormone levels and balanced the relative abundance of beneficial and harmful bacteria and the gut microenvironment to promote digestion and absorption of contents in the intestine. QR facilitated elimination of the contents by increasing the water of the contents via regulating gastrointestinal hormones, which also facilitated the inward flow of Ca^2+^ through the CAM-MLCK pathway, thus enhancing gastrointestinal motility levels. Furthermore, QR enhances the abundance of gut microbiota, restores the neuro-neurotransmission of the enteric nervous system, and promotes intestinal motility. This study provided an experimental basis for the compatibility of QR and BBR and might provide a new paradigm for the research and development of new drugs as well as dosage forms for the clinical combination applications of QR.

## Data Availability

The original contributions presented in the study are included in the article.
